# Fungus Hæmatodes

**Published:** 1828-12

**Authors:** 


### FUNGUS HÆMATODES.

### *Amputation of a large Fungus Hæmatodes of the Breast.*

Mrs. T., aged thirty-five, ten years ago first perceived a tumor, about the size of a walnut, in her right breast, which gave her no pain, and did not sensibly increase until she weaned her second child, two years and a half ago. From that period it enlarged rapidly, and when she came to Gloucester to consult Mr. Fletcher, (some time in October 1827,) it had attained a very considerable size. The growth of the tumor was attended with a dull heavy pain: it was likewise subject to periodical attacks of active inflammation, during which shooting pains were experienced, and the glands in the axilla became temporarily swollen. Mr. Fletcher recommended its removal, to which she would not then consent. Recourse was therefore had to the repeated application of leeches, with low diet, which diminished the tumor for a time, but produced no permanent benefit.

The tincture of iodine was tried at one time, and at another a course of mercury, without any perceptible advantage.

She then left Gloucester for some months, and returned the beginning of July, 1828, to the Gloucester Infirmary, having determined to submit to an operation.

At that time the tumor had attained a very large size, and appeared to consist of several masses, having a firm and somewhat elastic feel. Veins, very numerous, and as large as the little finger, ramified in all directions over its surface. She complained of a dull heavy pain, but no shooting or lancinating pains were experienced. There was a slight adhesion near the sterno-clavicular articulation. The glands in the neighbourhood of the disease were neither enlarged nor painful.

The remarkable size of the tumor, its progress, history, and strongly vascular character, decided Mr. Fletcher in the opinion that the complaint was of the nature of fungus hæmatodes. A vast hemorrhage was therefore anticipated on its removal, and, as the shock of so severe an operation would inevitably require all the powers of the constitution to withstand it, the least unnecessary loss of blood was to be guarded against with more (if possible) than usual care.

Hence Mr. Fletcher directed that, immediately on the removal of the tumor, two of the attendants should be ready to place the palms of their hands firmly on the bleeding surface. The subclavian artery was compressed over the first rib, by means of a large padded key. Three or four ounces of wine were given before the commencement of the operation.

The first incision was quickly made round the tumor, wide of its base, when some arteries bled furiously, one of which was tied; but as it was thought that the advantage of securing the rest would not compensate for the loss of blood, the dissection was rapidly proceeded with, and the breast removed, by the knuckles and the knife, in about a minute and a half. The rush of blood, chiefly venous, was tremendous, and, though commanded as soon as possible, by the means previously directed, the loss was very considerable, probably not less than three pints. The pressure on the sore, and that on the subclavian, were continued for eight or ten minutes, when the former being gradually removed, the vessels which bled (about seven in number) were secured.

The shock produced on the nervous system by this severe operation, and the great loss of blood, reduced the powers of life to a very low ebb. The patient was cold, livid, and speechless. Great quantities of wine and brandy were given during the operation, and some at intervals afterwards; and, until five hours had elapsed, it was not thought safe to remove her off the table into her bed.

The parts removed weighed exactly *seven pounds and a quarter*, and, the diseased structure being cut into, exhibited the usual brainlike appearances.

Abundance of skin might have been saved in this operation, but Mr. Fletcher determined to keep the wound quite open, that he might thus be enabled to detect any unhealthy appearances; and it was fortunate that this plan of treatment was adopted, for, during the first five weeks, small vascular spots, with yellow lymph deposited round them, were continually presenting themselves in different parts of the surface, and were immediately destroyed by the strong arsenical ointment, or nitric acid very slightly diluted. For the last month nothing of the kind has been seen, and the wound is contracted to the size of a crown-piece, with a healthy granulating even surface, presenting altogether a prospect most favorable and satisfactory.

